# How not to put the O into an OSCE

**DOI:** 10.1007/s40037-018-0424-y

**Published:** 2018-04-24

**Authors:** Tim Wilkinson

**Affiliations:** University of Otago, Christchurch, New Zealand

**Keywords:** Objective structured clinical examination, Reliability

## The story

When I was a younger academic, our medical school set out to improve the assessment of medical students. We had a series of in-training assessments, but the main end of year assessments consisted of essay-style questions. The suggestion was made to change the end of year exam to a multiple choice written examination and an objective structured clinical examination (OSCE) of 20 five-minute stations. The in-training assessments would become formative and not count towards deciding a student’s progression. While I did not mourn the loss of essay-style questions, I was worried that the replacement of in-training assessments with an OSCE might introduce a degree of artificiality. I was also worried about the logistic implications of mounting a large-scale OSCE, compounded by our course running over three cities meaning we had to offer the same OSCE simultaneously in three places. One of the arguments for introducing the OSCE was because of its objectivity. Striving to achieve such objectivity was where my young and naïve mistakes emerged.

There were many meetings when these proposals were discussed and I suggested that a better way might be to make more use of the existing in-training assessments, which could perhaps be streamlined better and then we need not worry about bringing in the OSCE.

In the midst of these discussions, I received a call from the Dean who thanked me for my insights and views which he found very useful. I was pleasantly surprised. He then informed me that he had decided to proceed with the proposal anyway and introduce the OSCE. In view of my interest in the area, he asked if I could be in charge of organizing this change? I was unpleasantly surprised. Of course, you could have seen that coming. What better way to deal with naysayers than to get them involved in devising the solution. I realize that now.

So there I was, early in my career, charged with a substantial logistic exercise. With a lot of help from friends and colleagues, we managed to produce a well-organized OSCE that has continued in an evolving form to this day [1]. I thought, if I am going to be in charge of this, I need to make sure it is of high quality.

One of the early decisions we made was to ensure there were two examiners for each station—on the grounds that this is a high stakes examination and the students deserve to know that it will be as robust as possible. In those days, my only concept of reliability (which I equated with ‘objectivity’) was to look at interrater correlations. After the first few iterations of the OSCE, and depending on the station and pairs of examiners, these correlations ranged from 0.37 to 0.90. This measure of agreement between examiners was modest at best and did not reassure us that they were ‘agreeing’ on what was intended. This contrasted with how much the stations seemed to be assessing the same thing—the internal consistency, or Cronbach alpha, which was respectable at 0.75 to 0.85. Nevertheless, I thought that surely we could do better on our interrater agreements?

My mission then became to look at ways to improve those interrater agreements. Objectivity had to relate to having clear criteria, right? Having clear criteria meant having better checklists, right? Having better checklists probably meant having more of them, maybe? These developments were occurring just at the time when we were learning about the difference between objectivity and objectification [2]. Objectivity has been expressed as a goal of measurement, marked by freedom of subjective influences, while objectification has been described as a set of strategies designed to reduce measurement error [2]. The problem with objectification strategies is that they risk trivializing the content being assessed. I was blissfully unware of all this at the time.

## Surprising outcomes

We included more checklists and found the interrater correlations did not improve—if anything they got worse [3]. The examiners complained bitterly—‘we’re spending so much time looking at these checklists, we hardly have any time to look at the student’.

## Lessons learned

We now know that reliability comes from making decisions after assimilating several observations and that the O in the OSCE comes not from the guise of objectivity inherent in checklists, but from the multiple observations. We know that now. We converted all those checklists into bullet points of ‘important things to look for in this station’. We left the examiners freedom to make global ratings. The examiners were happy, the reliability improved. Because the interrater agreement and internal consistency both improved, we have been able to move to fewer, but longer, stations and thereby increase the authenticity of what is assessed within each station. Ironically current discussions in assessment focus upon how we might make greater use of in-training assessment data—which was where I started in this journey.

## Moral of the story

Even today, people often focus on trying to tie down the minutiae in assessment forms, instead of trusting the global judgements of experienced raters. I have learnt that the considered judgments by many people, when taken together, can create value that we can trust—sometimes referred to as the wisdom of the crowd [4] or the subjective collective [5]. These are now familiar concepts. Moral 2—be careful what you complain about—you might just be asked to fix it.
